# Medical Device-Associated Infections Caused by Biofilm-Forming Microbial Pathogens and Controlling Strategies

**DOI:** 10.3390/antibiotics13070623

**Published:** 2024-07-04

**Authors:** Akanksha Mishra, Ashish Aggarwal, Fazlurrahman Khan

**Affiliations:** 1 School of Bioengineering and Biosciences, Lovely Professional University, Phagwara 144001, Punjab, India; akanksha.42200033@lpu.in; 2 Institute of Fisheries Science, Pukyong National University, Busan 48513, Republic of Korea; 3 International Graduate Program of Fisheries Science, Pukyong National University, Busan 48513, Republic of Korea; 4 Marine Integrated Biomedical Technology Center, The National Key Research Institutes in Universities, Pukyong National University, Busan 48513, Republic of Korea; 5 Research Center for Marine Integrated Bionics Technology, Pukyong National University, Busan 48513, Republic of Korea

**Keywords:** biofilms, nosocomial infections, healthcare-associated infections, antibacterial, antifouling, surface coatings

## Abstract

Hospital-acquired infections, also known as nosocomial infections, include bloodstream infections, surgical site infections, skin and soft tissue infections, respiratory tract infections, and urinary tract infections. According to reports, Gram-positive and Gram-negative pathogenic bacteria account for up to 70% of nosocomial infections in intensive care unit (ICU) patients. Biofilm production is a main virulence mechanism and a distinguishing feature of bacterial pathogens. Most bacterial pathogens develop biofilms at the solid-liquid and air-liquid interfaces. An essential requirement for biofilm production is the presence of a conditioning film. A conditioning film provides the first surface on which bacteria can adhere and fosters the growth of biofilms by creating a favorable environment. The conditioning film improves microbial adherence by delivering chemical signals or generating microenvironments. Microorganisms use this coating as a nutrient source. The film gathers both inorganic and organic substances from its surroundings, or these substances are generated by microbes in the film. These nutrients boost the initial growth of the adhering bacteria and facilitate biofilm formation by acting as a food source. Coatings with combined antibacterial efficacy and antifouling properties provide further benefits by preventing dead cells and debris from adhering to the surfaces. In the present review, we address numerous pathogenic microbes that form biofilms on the surfaces of biomedical devices. In addition, we explore several efficient smart antiadhesive coatings on the surfaces of biomedical device-relevant materials that manage nosocomial infections caused by biofilm-forming microbial pathogens.

## 1. Introduction

There is a high incidence of nosocomial infections caused by contaminated medical equipment, such as urinary catheters, intravascular catheters, and orthopedic implants, which contain pathogenic bacteria [1]. These pathogens include methicillin-resistant *Staphylococcus aureus*, *Escherichia coli*, *Acinetobacter baumannii*, and *Pseudomonas aeruginosa* [2]. Healthcare-associated infections (HAIs) are a global occurrence that leads to significant increases in mortality and morbidity. These present serious issues in both underdeveloped and highly developed European countries [3]. Numerous studies have shown that the types of bacteria that cause HAIs vary depending on the type of medical implant the patient has [4]. Accurate determination of whether the patient contracted the bacteria before hospital admission or during the hospital stay is crucial. Only infections that manifest in patients 48 h after admission are classified as HAIs. Symptoms, including fever, chills, fatigue, coughing, dyspnea, stomach pain, and loose stools, indicate that the patient is infected. Inflammation and sepsis are typical symptoms [5]. Catheterization-related nosocomial infections are often linked to antibiotic-resistant microorganisms, such as *Staphylococcus*, *Enterococcus*, and different enterobacterial species, as well as fungi, such as *Candida* spp. [6]. Approximately 40,000 hospitalized patients die each year globally from HAIs, which account for over 25% of the ailments attained during the process of health care in developing countries and up to 15% in wealthy countries [7]. Infections with different microorganisms (including *P. aeruginosa*, *Klebsiella pneumoniae*, *A. baumannii*, and *E. coli)* can cause bloodstream infections, which are a primary cause of death, prolonged hospital and Intensive Care Unit (ICU) stays, and increased healthcare costs [8]. The majority of indwelling central venous catheters are colonized by microorganisms embedded in a biofilm matrix, as demonstrated by scanning electron microscopy [9].

**Figure 1:**
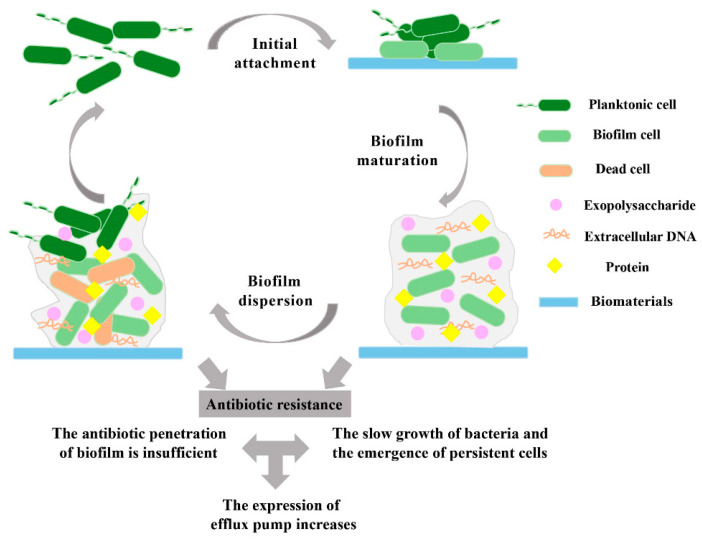
Stages of biofilm formation and mechanism showing antibiotic resistance by pathogenic bacterial strain. Reprinted from the [19], Copyright © 2023 by the authors and Licensee MDPI, Basel, Switzerland.

Research has demonstrated that bacterial adhesion and biofilm formation can occur in various medical devices, including dental chair water lines, indwelling stents, urinary catheters, intrauterine devices, and contact lenses [10]. There are multiple stages in the establishment and growth of biofilms; single bacterial attachment (both reversible and irreversible), bacterial aggregation, microcolony formation, maturation, and dispersion/detachment are the five main stages of bacterial attachment [11] (Figure 1). Quorum sensing (QS), a key mechanism in bacterial communication, involves the production, release, detection, and response to extracellular signaling molecules [12,13]. QS signaling enables bacteria to collectively modify their behavior, including the production of virulence factors and biofilms, in response to changes in cell density and community composition [12,14]. Since the beginning of time, bacteria have been on Earth in two states. Sessile bacteria are said to be 500–5000 times more resistant to antibiotics than their planktonic counterparts [15]. The complex, multi-step, and usually cyclic process of biofilm formation involves several bacterial species. Bacterial biofilms secrete extracellular polymeric material, which is a mixture of polysaccharides, proteins (mostly composed of D-amino acids), fatty acids, and different nucleic acids [16]. According to the National Institutes of Health, biofilms can be responsible for up to 80% of human microbial infections. These infections include meningitis, kidney infections, endocarditis, cystic fibrosis, periodontitis, rhinosinusitis, osteomyelitis, non-healing chronic wounds, and infections related to prosthetic and implantable devices [17]. The microbiological factors that control the development of biofilms have been identified by in vitro analyses of biofilm infection [18].

**Figure 2:**
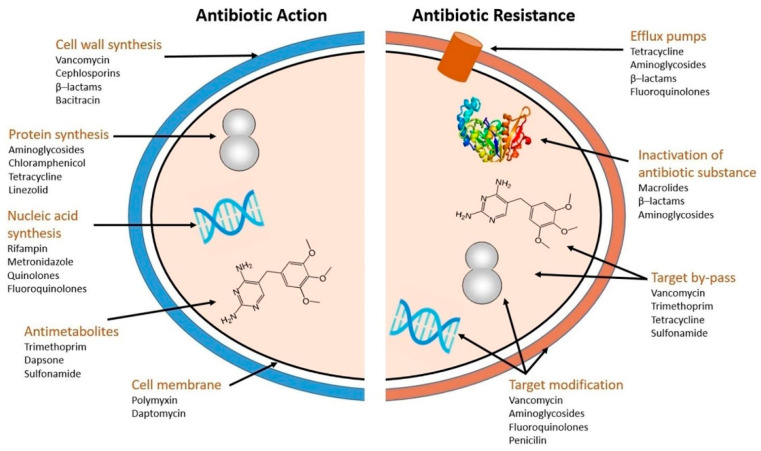
Mechanisms of resistance to antibiotics and their effects. Reprinted from the [27], Copyright © 2021 by the authors and Licensee, Frontiers in Microbiology (Lausanne. Switzerland).

The development of biofilms on the surfaces of medical devices is a major contributing factor to the infections associated with these devices [20,21]. These biofilms provide a protective barrier for bacteria, rendering them resistant to antimicrobial treatments and increasing the risk of infection [19]. At least three intricate components are involved in microbial colonization: the device, microbes, and the host environment (such as tissues and immune cells). The surface properties of medical implants, such as their chemical composition and morphology, play a crucial role in biofilm formation and bacterial adhesion [22]. Microbial colonization is frequently difficult to identify. In certain situations, it may go unnoticed for years; however, in other situations, it may be urgent enough to endanger life [23]. Slimy “biofilm” coatings created by invading bacteria have been found on a number of gadgets that were discarded following problems due to microbial colonization [24]. Recalcitrance, a biofilm lifestyle trait that causes medication failure and infection recurrence, refers to the ability of pathogenic biofilms to thrive in the presence of high doses of antibiotics [25]. The word “recalcitrance” refers to a subgroup of biofilm-forming bacteria that can survive in the presence of high doses of antibiotics (Figure 1). The distinct adaptive antimicrobial resistance mechanism known as biofilm-forming bacteria is directly responsible for a number of therapeutic problems that arise in clinical settings and ultimately result in the death of patients [26]. It encompasses the idea of non-susceptibility to (antimicrobial) control of refractory biofilms [25]. Although bacteria have multiple antibiotic resistance mechanisms, such as target modification, efflux pump expression, inactivation of antibiotic substances, and target bypass [27] (Figure 2), the formation of biofilms exhibits an adaptive resistance mechanism against antibiotics, as well as bypassing the host defense systems [28]. The biofilm matrix also prevents antibiotics from reaching the cells, contributing to the overall resistance [29]. This structure also facilitates the flow of antimicrobial-resistant genes between and within the species, protects against drug penetration, and increases their persistence [30].

The formation of a biofilm when bacteria contaminate an indwelling medical device depends on several factors [31]. Before permanently connecting, the microbes must cling for a sufficient time to expose the tool surfaces. The type and number of cells influence the rate of cell attachment in the liquid to which the device is exposed, the liquid flow rate through the device, and the physicochemical characteristics of the surface [11]. It is possible for liquid components to alter the surface properties as well as the pace of adhesion. The flow rate, nutritional composition of the medium, concentration of the antimicrobial medicine, and ambient temperature affect the pace of growth of these cells once they irreversibly attach to and generate proteins found outside the cells to create a biofilm [32]. Biofilms formed on three different types of indwelling medical devices were used to illustrate these variables: central venous catheters, urine (Foley) catheters, and mechanical heart valves [33]. Different stages of biofilm adherence to the surface of medical devices have been reported previously [34]. To strengthen the knowledge of possible strategies to control biomedical device-associated infections, the present review aims to discuss (1) the clinical significance of medical device-associated infections caused by biofilm-forming bacterial pathogens and (2) possible treatment strategies for controlling biomedical devices associated with biofilms using different materials.

## 2. Role of Biofilms in HAIs

The most prevalent biofilm-based illness caused by medical equipment is catheter-associated urinary tract infection (CAUTI), which affects over 150 million individuals worldwide annually [35]. Flexible multichannel endoscopes are a unique type of reusable medical device. If reprocessing guidelines are not followed properly, biofilm growth may occur. Biofilm production thrives in damp, nutrient-rich conditions inside the lumen of an endoscope used on a patient [36]. A protein-containing coating is formed around an implant or other device when it enters the body, facilitating bacterial colonization. After bacteria attach to themselves, biofilms begin to form. When bacteria reach maturity, they begin to spread, and some enter the bloodstream, potentially leading to serious infections [37]. Following a thorough examination of the data regarding bacterial adherence and device surface change, the following five main principles are identified: (1) Different bacteria could attach to the same device material in various ways; (2) the same bacteria may attach to various device materials in different ways; (3) bacteria may attach differently to the same device material under different environmental conditions, such as the type of flow (stationary vs. dynamic), temperature, and the hydrophobic versus hydrophilic medium in which the device is placed; (4) the prevention of bacterial colonization of the device in vitro cannot ensure anti-infective effectiveness in vivo; and (5) depending on the application, different surface-modifying approaches may have different therapeutic benefit [38].

## 3. Pathogenesis of Biofilm-forming Microbes

Bacterial biofilms are prevalent in the human body and can have significant impacts on health and disease [39]. Biofilms have been shown to grow on the surface of medical equipment, and the distribution of both single and clustered cells implies a substantial risk of microbial dissemination within the host and an elevated risk of infection. Hospitals, assisted living facilities, and even patients’ homes can have bacteria that cause HAIs [40]. Perry and Tan [39] summarized the formation of bacterial biofilms in the human body at different locations, such as the upper respiratory tract, middle ear, soft tissue wounds, urinary tract, male and female reproductive tract, bone, oral cavity, cardiovascular system, stomach, and colon.

The human oral cavity initially creates an aerobic environment where oxygen is first consumed by facultative anaerobic bacteria (such as *Actinomyces* and *Streptococcus* spp.) or aerobic bacteria (such as *Neisseria* spp.), creating an environment that is suitable for the survival of obligate anaerobic bacteria. Anaerobic bacteria predominantly populate the human oral cavity during biofilm formation [41]. When wound healing is disrupted, exogenous infections usually occur during surgery or early postoperatively. Patients with large hematomas typically present with this condition. Exogenous infections rarely develop late in the healing process during arthrocentesis or after device-induced or spontaneous skin rupture [42]. Spontaneous skin rupture occurs more frequently after osteosynthesis than after joint replacement [43].

Biofilms have been found in the circulatory system of atherosclerotic arteries and in heart infections (endocarditis). When bacteria, most frequently *S. aureus*, *Streptococcus* species, and *Enterococcus* species, adhere to the heart valves or the inner lining of the heart chambers, they can cause infectious endocarditis (Table 1). This condition usually affects patients with congenital valve defects or damaged heart tissue [44]. Recent data imply that planktonic bacteria have long been associated with acute respiratory illnesses, where aggregate-type biofilms in sputum are commonly thought to be the predominant form, as well as chronic lung infections, as is widely acknowledged [45]. 

The incidence of infections linked to medical device biofilms has become a significant clinical issue because biofilms are resilient and resistant to antimicrobial therapy, and many researchers are now focusing on the mechanisms by which they form and thrive using standard antibacterial techniques [46]. Locally acquired host defense deficiency is the primary cause of implanted devices that increase sensitivity to infection, and the rapid development of a biofilm that is resistant to both host defense and antimicrobial treatments is the main cause of persistence [47]. Patients in the ICU have a heightened vulnerability to device-associated nosocomial infections owing to their compromised immune systems and frequent contact with invasive medical equipment. The use of these devices increases the incidence of ventilator-associated pneumonia (VAP), central line-associated bloodstream infections (CLABSIs), and CAUTIs in patients admitted to the ICU [48].

**Table 1:** Various medical devices and common pathogens form biofilm.

Medical Device	Common Pathogen	References
Orthopedic devices	Staphylococci, Gram-negative bacilli	[49]
Endotracheal tubes	*Pseudomonas aeruginosa*, *Staphylococcus aureus*	[50]
Contact lenses	*Pseudomonas aeruginosa*, Staphylococci	[51]
Intravascular catheters	Staphylococci, Enterococci, Gram-negative bacilli	[52]
Valves, pacemaker	Staphylococci, Streptococci	[53]
Respiratory equipment, Indwelling catheters	*Acinetobacter baumanii*	[54]
Bronchoscopy	*Klebsiella pneumoniae*	[55]
UTIs devices, Intravascular medical device	*Enterobacter* spp.	[56]
Respiratory device	*Aspergillus fumigatus*	[57]
Cardiac medical device	*Cryptococcus neoformans*	[58]
Urinary catheters	*Enterococcus faecalis*	[59]
Catheters, prosthetic devices	*Aspergillus fumigatus*	[60]
Central venous catheter	*Staphylococcus epidermidis*	[31]
Cerebrospinal shunts	*Staphylococcus aureus*, *Propionibacterium*	[61]
Dental implants	*Prevotella intermedia*, *Actinobacillus*	[62]

## 4. Establishment of the Biofilm on Biomedical Device Surfaces 

The challenges in employing available antibiotics to treat biofilm-associated illnesses, especially implant-associated infections, are exacerbated by bacterial biofilm tolerance and resistance. In 36 countries in Latin America, Asia, Africa, and Europe, 422 ICUs were examined by the International Nosocomial Infection Control Consortium. The results indicated 7029, 6595, and 12,145 cases of CLABSIs, CAUTIs, and VAPs, respectively, between January 2004 and December 2009. Implant-associated infections have gained attention because of the rapid development of implantable biomedical devices [63]. Significant increases in economic losses, morbidity, and mortality are linked to HAIs, many of which are unavoidable. The most frequent hospital-acquired infections are surgical site-and device-associated HAIs, including VAP, CLABSIs, and CAUTIs [64]. 

**Figure 3:**
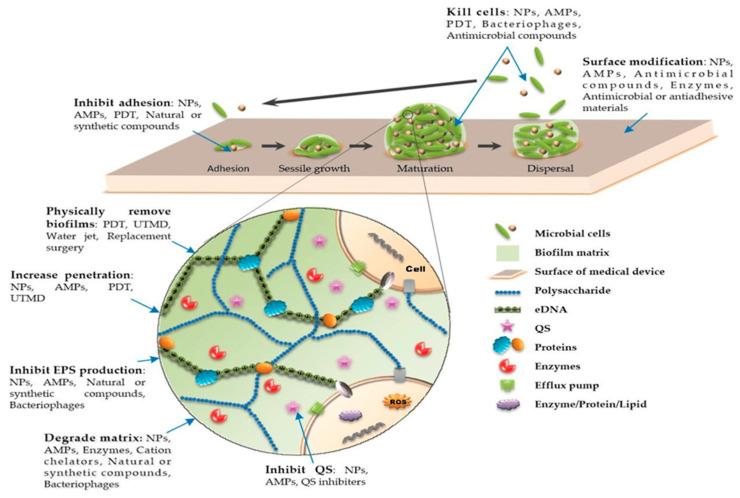
Stages of biofilm formation on the surface of the medical devices. Reprinted from the [73], Copyright © 2021 by the authors and Licensee MDPI, Basel, Switzerland.

Furthermore, infections are common and frequently associated with surfaces and devices that have biofilms established on them [65]. Physiological gradients, matrix diffusion restrictions, and innate and evolved resistance mechanisms make it challenging to control biofilms formed on medical devices using antimicrobials. Together, these factors promote antimicrobial resistance [66]. Cell adhesion, which is required for biofilm development and other biological processes, is an essential component of interactions between cells and surfaces. Surface wettability, rather than polymer type or surface topography, is a key factor that influences cell attachment and proliferation [67]. Biofilm formation initiates the disease process through various mechanisms, including the detachment of individual bacterial cells or clusters of cell aggregates, the production of endotoxins, increased evasion from host immune system surveillance, and the creation of a protective barrier that fosters the emergence of immune-resistant organisms (Figure 3). When pathogens, such as *P. aeruginosa*, attach themselves in real time to biomedical equipment in the clinic, biofilm formation can be monitored. This allows timely antibiotic treatment or device removal when the first signs of bacterial attachment are observed [68]. By doing this, the maturation of biofilms can be stopped, and chronic infections and related difficulties can be lessened [69]. Early action can stop biofilms from growing until they reach the dispersion stage of their life cycle, leading to systemic infections. This strategy would reduce healthcare costs for each patient while improving their health. Currently, symptoms are used to detect device-related infections. However, to examine the developed biofilm, the device must be disassembled for microscopic and microbiological examinations [70].

This laborious procedure postpones appropriate corrective measures to prevent the worsening of the infection [71]. When a biofilm forms on a medical device, removing the bacteria can be difficult and expensive because extensive hospital stays, surgeries, and long-term antibiotic treatments are frequently required [72]. It is widely acknowledged that biofilm formation is one of the key virulence factors in infections linked to medical devices [21].

## 5. Recent Advancements in Antiadhesive or Antifouling Coating on the Surface of Biomedical Devices

**Figure 4:**
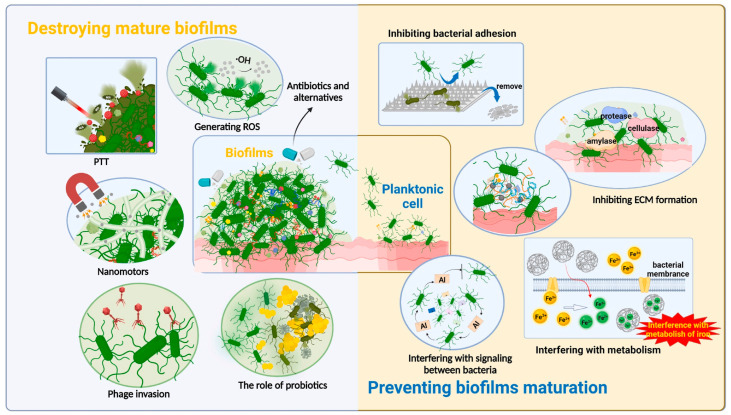
Representation of biofilm formation control and its removal by various methods. Reprinted from the [95], Copyright © 2023 by the authors and Licensee, Elsevier Ltd. (Amsterdam, The Netherlands).

The development of a promising antibacterial coating on the surface of biomaterials and biomedical devices is facilitated by the integration of bactericidal and antifouling coatings, as both functions work synergistically to provide benefits [74]. The most popular antimicrobial strategy involves the addition of bactericidal materials or reagents to a surface and carrying out antimicrobial activities through release or contact killing, which are referred to as releasable and non-releasable bactericidal compounds, respectively [75]. To effectively prevent microbial biofilms and biological biofouling infections caused by biomaterials and biomedical devices, they must demonstrate not only superior growth-inhibitory efficacy against pathogenic bacterial species but also the capacity to prevent the adhesion of live or dead microbiological species and nonspecific platelets, proteins, and other biological macromolecules [76]. By reducing the adhesion force between a solid surface and bacteria, antiadhesive/antifouling surfaces enable bacteria to be readily removed before a biofilm develops [77]. Barnacle cement has been successfully used as a surface anchor to attach antifouling and antimicrobial polymer brushes to stainless steel [78]. This approach has been shown to be stable and effective, reducing protein adsorption and bacterial adhesion [79]. Other studies have also explored the use of biomimetic anchors, such as polydopamine layers, for the attachment of functional polymer brushes to stainless steel, resulting in enhanced antifouling and anticorrosion properties [80]. A one-step anchoring method using tannic acid-scaffolded bifunctional coatings has also been developed, further improving resistance to protein adsorption and bacterial adhesion [81]. Recent studies have made significant strides in the development of antibacterial coatings to combat orthopedic implant-associated infections (Figure 4). Wang et al. [82] and Akay and Yaghmur [66] highlighted the potential of antibacterial hydrogel coatings and modified implant surfaces to prevent biofilm formation. These coatings are designed to inhibit bacterial attachment and colonization, thereby reducing the risk of infection. Wang et al. [83] discussed the use of biodegradable alloy materials with inherent antibacterial properties as orthopedic implant materials, thereby providing a promising alternative to traditional implants. However, the need for further research and clinical testing of these coatings was emphasized by Wang et al. [83], underscoring the importance of continued advancements in this field.

Several studies have demonstrated the application of antibacterial coatings consisting of nanoparticles and lipids to minimize implant-related infections [84,85,86]. Nanoparticles and lipid coatings play crucial roles in enhancing the performance of biomedical devices. Both Luchini et al. [87] and Simović et al. [86] highlight the potential of lipid-coated inorganic nanoparticles in improving the stability, performance, and biocompatibility of lipid-based colloids, as well as in drug delivery systems. Jiménez-Jiménez et al. [88] further emphasized the versatility of this technology, particularly in the use of cell membranes to coat nanoparticles, which can improve their performance in various applications. Mashaghi et al. [89] underscore the significance of lipid nanotechnology in these advancements, particularly in the fields of targeted drug delivery and bioimaging. These studies collectively demonstrate the potential of nanoparticles and lipid coatings to enhance the functionality and effectiveness of biomedical devices (Table 2). Numerous types of metal nanoparticles have proven to be effective antibacterial agents [90,91,92,93]. The most frequently used antibacterial nano-agents are oxide-based nanoparticles of silver, gold, copper, titanium, nickel, magnesium, and zinc [94].

**Table 2:** Different types of antiadhesive or antibiofilm coating agents are employed on the biomedical device surface.

Name of Biomedical Devices	Antibacterial Materials	Antiadhesive or Antibiofilm Coating Agents	Types of Pathogens	Efficacy (Killing of Attached Cells in %)	References
Polydimethylsiloxane (PDMS), stainless steel (SS) surface	Antibacterial surfaces that are smart and photothermally activated	Tannic acid(TA) and Fe^3+^ ion	*Escherichia coli*	>99	[96]
Blood-contacting medical devices	metal–phenolic and catecholamine	CuII–GA/CySA coatings copper ions (CuII)-gallic acid (GA)/ cystamine (CySA)	*Escherichia coli*, *Staphylococcus aureus*	∼99	[97]
Surfaces made of a variety of materials, including silicone, glass, poly(methyl methacrylate) (PMMA) plates, metal surfaces, polypropylene fiber, and filter paper	Electrostatic attraction, physically rupturing cell walls	Coating with positively-charged Zeolitic imidazolate frameworks(ZIF) nano-dagger arrays	*Staphylococcus aureus*	NA	[98]
Silicon (Si), PDMS, and SS	Gold nanoparticle layer(GNPL)	Regenerable smart antibacterial surfaces	*Escherichia coli*	>99	[99]
Implantable device	Hydroxyethyl methacrylate(HEMA) and quercetin(Qe)	Dual-functional anti-biofilms surface	*Pseudomonas aeruginosa*, *Staphylococcus aureus*	NA	[100]
Metal materials surfaces	Poly (2-hydroxyethyl methacrylate) hydrophilic polymer with Quaternary ammonium salt(QAS)	Intelligent composite material surface with titanium content	*Staphylococcus aureus*, *Escherichia coli*	99.86 and 97.08	[101]
Biomedical catheters	Sulfamethoxazole (SMZ) and trimethoprim (TMP)	Polyethylene glycol(PEG)	*Staphylococcus aureus*, *Escherichia coli*	80 (*E. coli* and *S. aureus* cells)	[102]
Urinary catheter	Poly(styrene sulfonate) (PSS), quaternary ammonium, H_2_O_2_ enzyme	New zwitterionic copolymers (PTMAEMA-co-PSPE) with different proportions of sulfobetaine	*Staphylococcus aureus*	60	[103]
Foley catheters	*α*-aminoisobutyric acid	Biocompatible amino acids	*Escherichia coli*, *Bacillus subtilis*	NA	[104]
Catheters, stents, and dialysis equipment	Silver nanoparticles	2-Methacryloyloxyethyl phosphorylcholine	*Escherichia coli*, *Escherichia coli* K-12	>99	[105]
Dental implant	Quaternized polyethyleneimine	poly(glycidyl methacrylate)	*Staphylococcus aureus*	95.6	[106]
Implant medical devices	Gold nanoparticles	built a mixed-metal-organic network nanocluster	*Escherichia coli*	NA	[107]
Soft contact lens	Polyelectrolytes	Diclofenac sodium salt, doxifloxacin hydrochloride, and chlorhexidine diacetate monohydrate	*Pseudomonas aeruginosa*, *Staphylococcus aureus*	NA	[108]
Dental implants	Zinc (Zn) nanoparticles Electrohydrodynamic deposition	Nanoparticles of hydroxyapatite (nHA) and zinc oxide (nZnO)	*Streptococcus* spp.	NA	[109]
Sinus Stents	Ciprofloxacin nanoparticle	Poly-l-lactic acid (PLLA), ciprofloxacin	*Pseudomonas aeruginosa*	NA	[110]
Bone implant devices	Combined vancomycin and Melittin	Chitosan, bioactive glass, and melittin	*Staphylococcus aureus*	NA	[111]
Orthopedic implants	Liposome-encapsulated photosensitizers (PS), IR780, and perfluorohexane (PFH),	Lecithin, cholesterol and PEGylated DSPE	*Escherichia coli*, *Staphylococcus aureus*	99.62 and 99.63	[112]
Subcutaneous implants	Nitric oxide (NO)	NO-releasing xerogel coatings of silicone rubber	*Staphylococcus aureus*	82	[113]
Biomedical Implants	Black phosphorus	Black phosphorus nanosheets with *N*,*N*′- dimethyl propylene urea (DMPU)	*Bacillus subtilis*	99.69	[114]
Implantable medical devices	Titanium, titanium binding peptides (TiBP)	Chimeric peptide (TiBP(S)1–3 and E14LKK/H14LKK motifs.)	*Streptococcus mutans*, *Staphylococcus* *epidermidis*, *Escherichia coli*	NA	[115]
Biomedical implants	Nanoparticles of (silver and zinc pyrithione, ZnP)	Silver-based organomodified layered silicate additives nanoclays	*Staphylococcus aureus*	NA	[116]

NA, Not available.

## 6. Conclusions and Future Perspectives

Antimicrobial coatings are widely used; however, the standards for these coatings are particularly strict in biomedical applications, which is the subject of this study. The most common methods involve the prevention of bacterial adherence and killing microbes through surface-associated mechanisms or coatings that emit antibacterial chemicals. To combat the growing resistance to conventional antibiotics, metal and metal oxide nanoparticles, as well as 2D nanomaterials, have provided innovative substitutes for antibiotic treatments of hospital-acquired illnesses linked to biofilms. To prevent medical implants linked to infections, antimicrobial-releasing coatings have undergone the most research. The search for innovative anti-infective biomaterials might raise reasonable hope for averting infection problems, which are connected to both long-term medical implant use and surgical treatment. Future developments may involve customized coatings depending on the requirements of specific patients or the microbiological environment, given variations in patient reactions to biomaterials. Customizing coatings to target particular bacteria known to cause issues in a given patient may improve the effectiveness of antibacterial strategies. Future research perspectives are recommended to detect biofilms on biomedical device surfaces and to prevent biofilm formation on the surface.

- Early biofilm detection will undoubtedly aid patient treatment and reduce costs. However, this is only possible if detection procedures and techniques are continuously improved, which might be accomplished with the use of artificial intelligence tools [117].
- Mixed-species biofilms exhibit notable differences in growth rate, gene expression, living habits, and structural appearance compared with those of single species. These differences are primarily manifested in enhanced biofilm metabolic capacity, resilience to environmental stress, and community-level signaling. Further studies on mixed-species biofilms are required [118].
- Mixed-species biofilms predominate in nature and are common in human hosts, such as the lungs and oral cavities of individuals with cystic fibrosis. Therefore, further studies are required to define the interactions within multispecies biofilms and the consequences of these interactions on biofilm community growth, makeup, and longevity [119].
- An antibiofilm or antiadhesive coating could be developed to prevent biofilm formation that works upon three lines of defense with antiadhesive, bactericidal, and anti-quorum sensing properties, adapting to the bacterial biofilm formation mechanism. Further research is required on such antibiofilm coatings [120].

## Data Availability

Not applicable.
